# Supplementary figures and images for: Complexome profile of Toxoplasma gondii mitochondria identifies divergent subunits of respiratory chain complexes including new subunits of cytochrome bc1 complex
Source: PLoS Pathog. 2021 Mar 2;17(3):e1009301. doi: 10.1371/journal.ppat.1009301 (PMC7987180; doi:10.1371/journal.ppat.1009301)

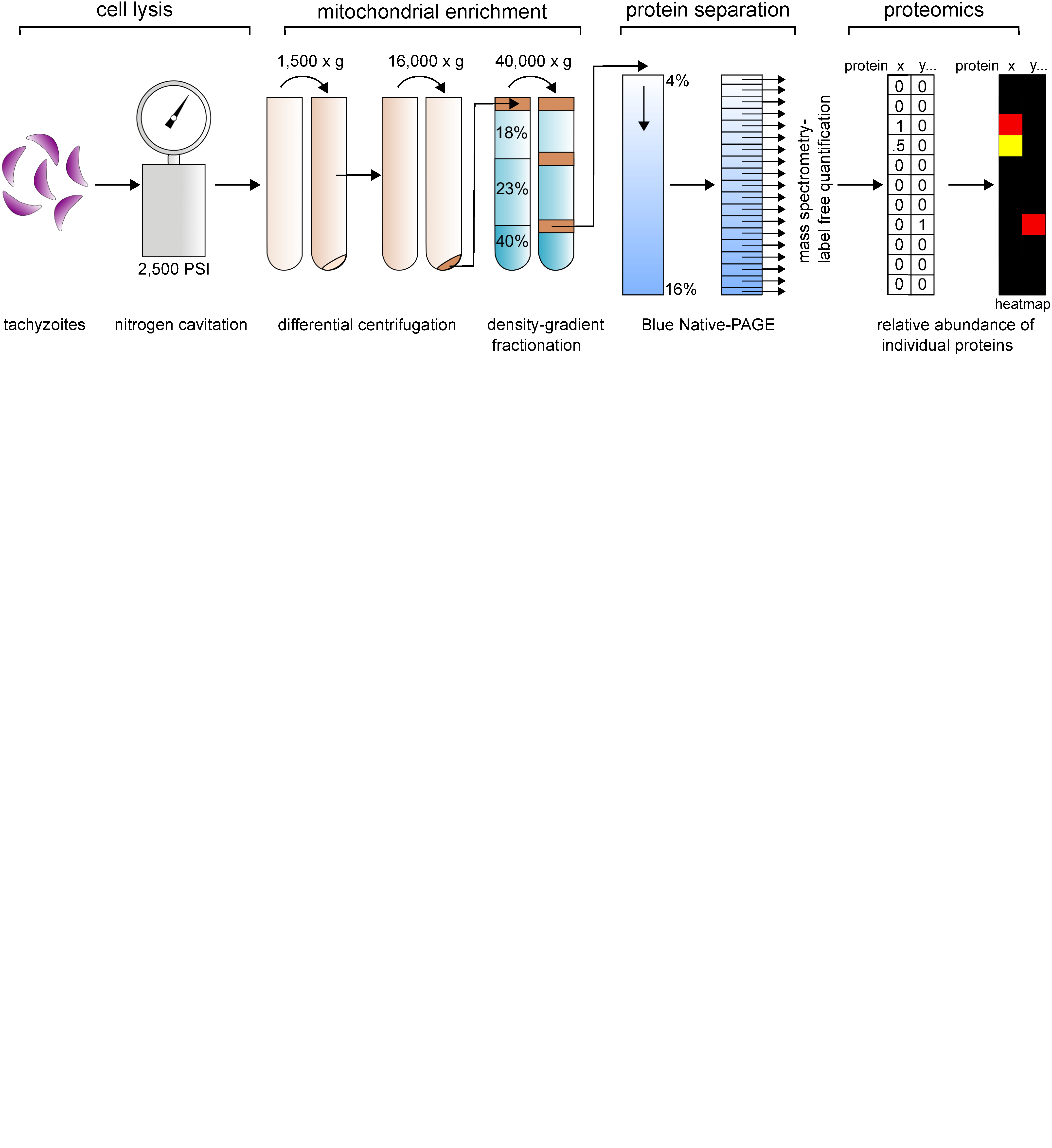

Supplement: S1 Fig — Schematic representation of the workflow for complexome profiling: a large number of Toxoplasma tachyzoites (~1 x 10^10^) were harvested and then cells lsyed using nitrogen cavitation. Three different centrifugation conditions were used to obtain a mitochondrially enriched fraction: differential centrifugation (at 1,500 x g and 16,000 x g) was used to separate organelles from unbroken cells and heavy cell debris. This fraction was then layered onto a Percoll step gradient and then, after density-gradient fractionation at 40,000 x g, recovered from the 23%/40% Percoll interface. Proteins were then separated on a Blue Native PAGE gel and the gel cut into 61 gels slices. Each gel slice was subjected to mass spectrometry and label free quantification performed. The resulting data allowed calculation of the relative abundance of individual proteins in each slice, and this is visualised using a heatmap. The total dataset gives the relative abundance information for hundreds of proteins across all gel slices. (TIF) [file ppat.1009301.s001.tif]

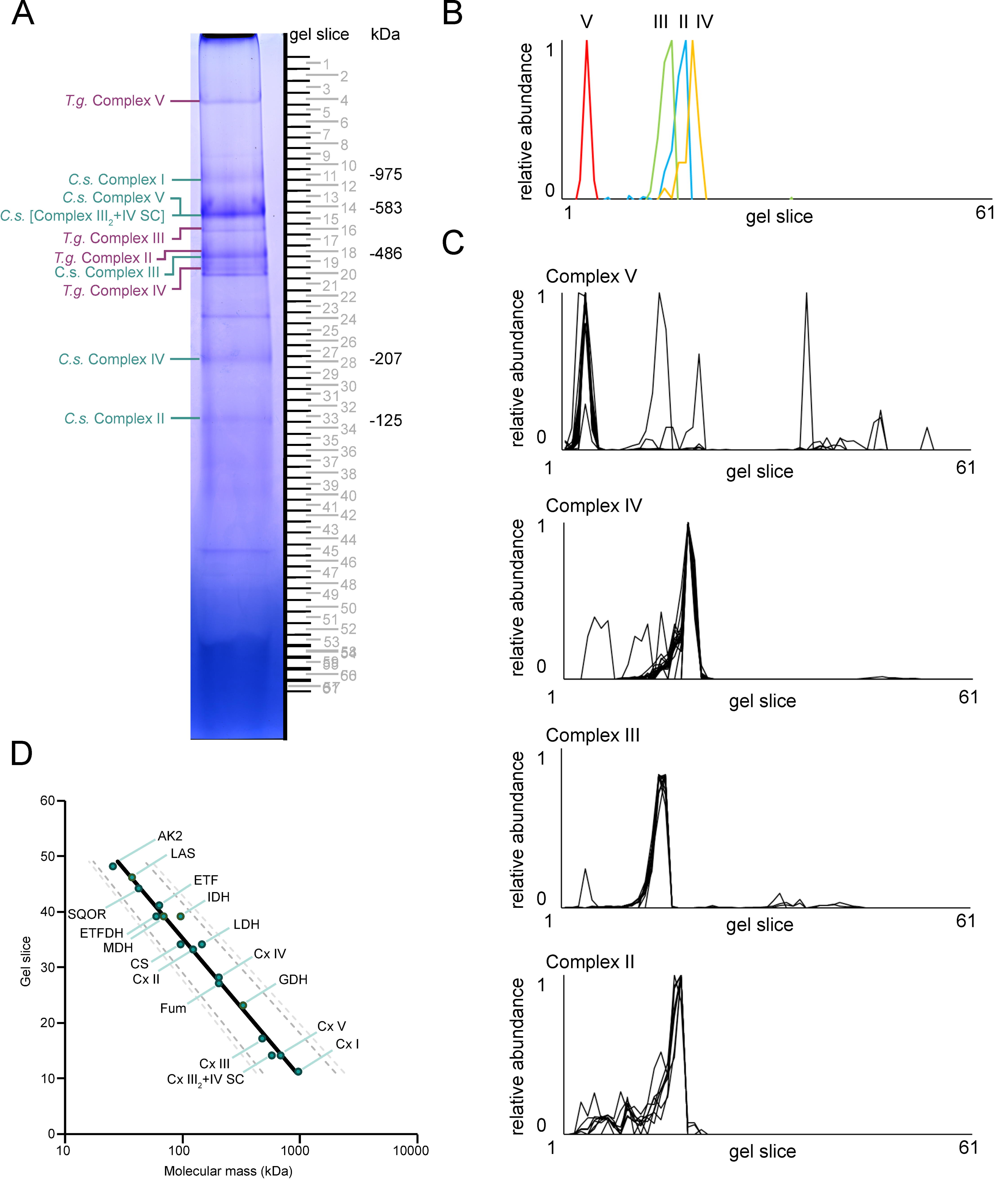

Supplement: S2 Fig — (A) Mitochondrial samples separated by blue native PAGE used to generate the complexome profile. The gel was cut into 61 gel slices as indicated on the right of the gel and molecular weights, based on the masses of mammalian complexes, indicated to the right of the gel slice numbers. The identities of host cell and Toxoplasma complex Coomassie-stained bands are indicated to the left of the gel strip. (B) Graphs depicting complexome profiles of T. gondii ATP synthase (V, red), complex IV, III and II (yellow, blue, green respectively) through a representative subunit from each complex (gamma for ATP synthase, Cox2a, Rieske and SDHB for complexes IV, III, II respectively). The x-axis depicts the gel slice number and the y-axis the protein’s relative abundance. The full complexome profiling dataset is provided in S1 Table. (C) Graphs depicting complexome profiles of all T. gondii subunits from ATP synthase (complex V), complex IV, III and II. The x-axis depicts the gel slice number and the y-axis the protein’s relative abundance. The full complexome profiling dataset is provided in S1 Table. (D) Mass calibration using C. sabaeus host cell complexes of known or predicted size. The x-axis depicts the molecular mass in kDa and the y-axis the gel slice number. Full detail of mass calibration is provided in S5 Table. AK2—Adenylate kinase 2; LAS—Lipoic acid synthetase; SQOR—Sulfide:quinone oxidoreductase; ETF—Electron-transferring flavoprotein A+B; ETFDH—ETF dehydrogenase; IDH—Isocitrate dehydrogenase; CS—Citrate synthase; Cx II—Complex II; LDH—Lactate dehydrogenase complex; Cx IV—Complex IV; Fum—Fumarase; GDH—Glutamate dehydrogenase; Cx III—complex III; Cx III2 +IV—Complex III + IV; Cx V—ATP synthase; Cx I—Complex I. (TIF) [file ppat.1009301.s002.tif]

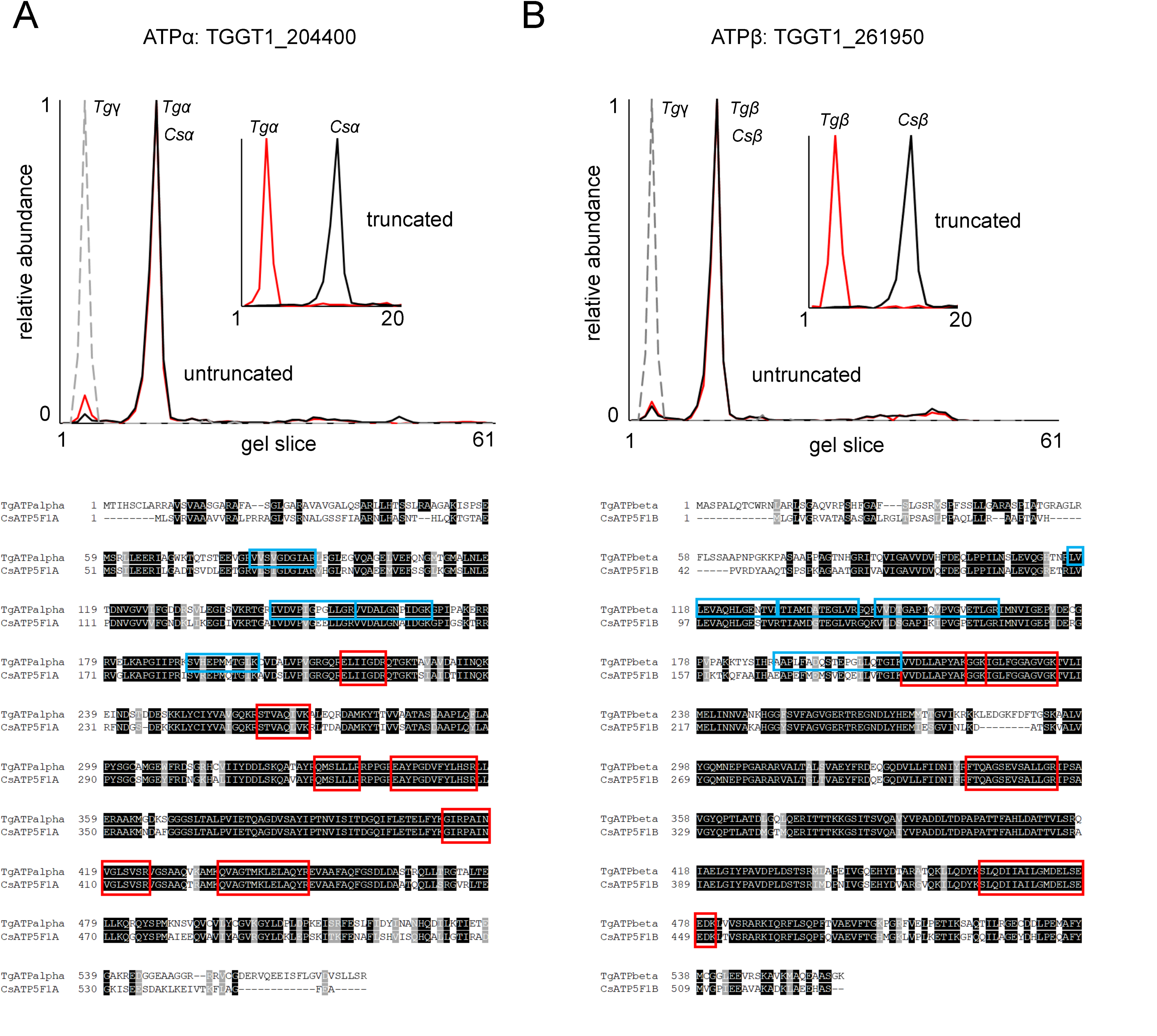

Supplement: S3 Fig — Graphs depicting complexome profiles and alignments of ATP synthase subunit alpha (A) and ATP synthase subunit beta (B) from T. gondii (red, Tgα and Tgβ) and C. sabaeus (black, Csα and Csβ) before and after truncation of common peptides. ATP synthase subunit gamma from T. gondii (grey, dashed line, Tgγ) depicts the position of T. gondii ATP synthase subunits. The x-axis depicts the gel slice number and the y-axis the protein’s relative abundance. Common peptides between the two proteins are outlined in the alignment by a red box, and Toxoplasma specific peptides after truncation are marked by a blue box. Alignments were performed by Clustal Omega. Full detail of protein truncation is provided in S6 Table. (TIF) [file ppat.1009301.s003.tif]

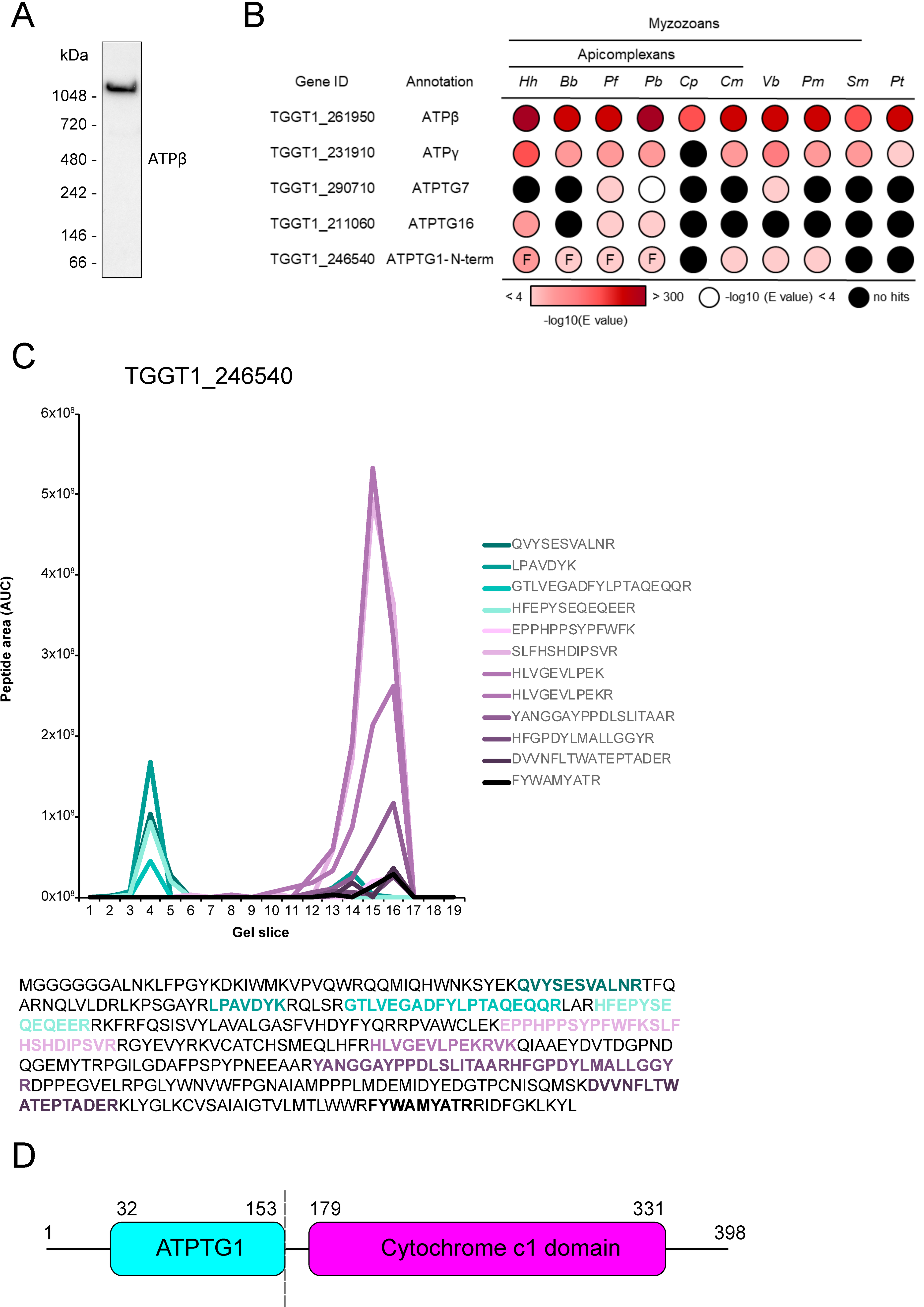

Supplement: S4 Fig — (A) Total lysate from parasite cells separated by BN-PAGE and immunolabelled with antibodies against the betta subunit of ATP synthase (ATPβ). (B) Table showing examples for two previously identified subunits—ATP synthase subunit beta and ATP synthase subunit gamma (ATPβ,γ) (known) and complexome identified putative novel ATP synthase subunits—ATPTG1,7,16 (novel) and their homology distribution across key groups. Homology searches were performed using the HMMER tool [57]. Coloured circles refer to the e-value from the HMMER search: white indicates a hit with an e-value above 0.0001, black indicates no hits, and red indicates hits with an e-value below 0.0001, as indicated in the coloured scale. Full data are given in S7 Table. Hh: Hammondia hammondi; Pb: Plasmodium berghei; Cp: Cryptosporidium parvun; Cyryptosporidium muris; Vb: Vitrella brassicaformis; Pm: Perkinsus marinus; Sm: Symbiodinium microadriaticum; Pt: Paramecium tetraurelia. (C) Graph depicting peptide abundances detected for TGGT1_246540 in the complexome profile and the amino acid sequence of the protein with the peptides detected in colour. Peptides from the N-terminal portion of the protein, which constitute ATPTG1 are marked in cyan, and peptides in the C-terminal portion of the protein, which constitute CytC1, are marked in magenta. (D) Schematic diagram of TGGT1_246540 showing the amino acids that make up ATPTG1 (cyan) and the cytochrome c1 domain (magenta). Residues in bold show the detected peptides. (TIF) [file ppat.1009301.s004.tif]

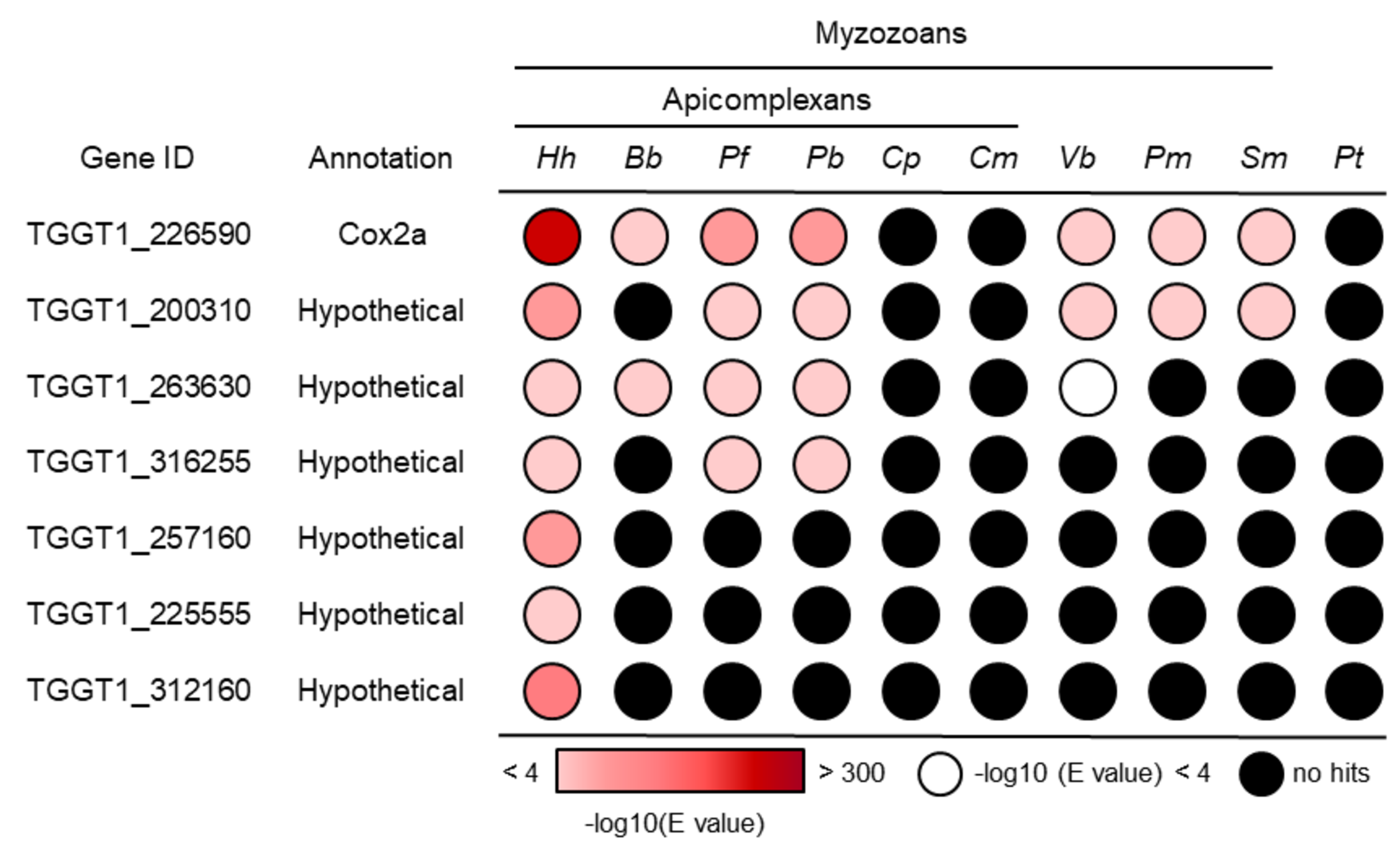

Supplement: S5 Fig — Table showing previous predicted (known) and complexome identified putative novel (novel) complex II subunits and their homology distribution across key groups. Homology searches were performed using the HMMER tool [57]. Coloured circles refer to the e-value from the HMMER search: white indicates a hit with an e-value above 0.0001, black indicates no hits, and red indicates hits with an e-value below 0.0001, as indicated in the coloured scale. Full data are given in S7 Table. Hh: Hammondia hammondi; Bb: Babesia bovis Pf: Plasmodium falciparum; Pb: Plasmodium berghei; Cp: Cryptosporidium parvun; Cyryptosporidium muris; Vb: Vitrella brassicaformis; Pm: Perkinsus marinus; Sm: Symbiodinium microadriaticum; Pt: Paramecium tetraurelia. (TIF) [file ppat.1009301.s005.tif]

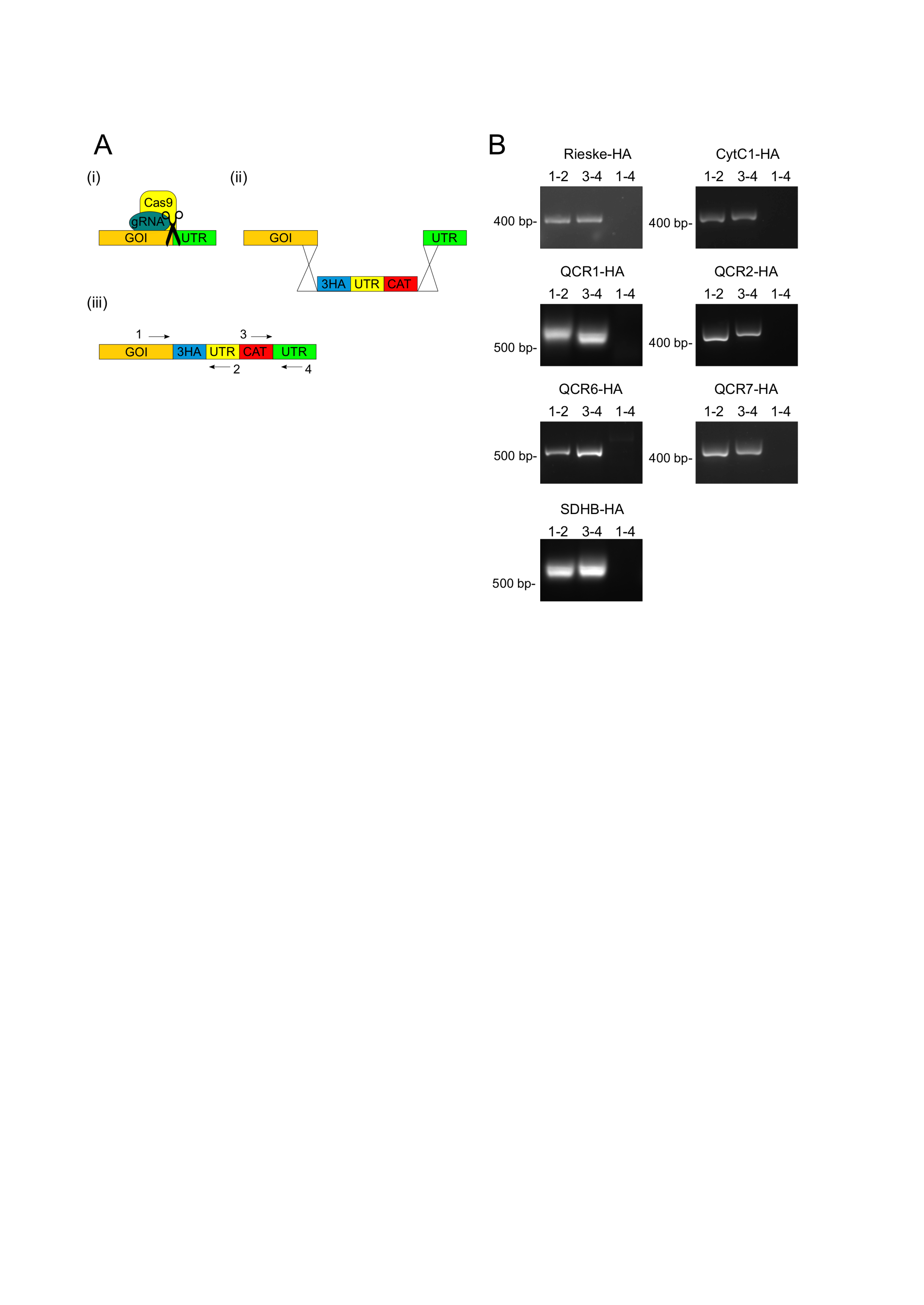

Supplement: S6 Fig — (A) Schematic depiction of the endogenous HA-tagging strategy of a gene of interest (GOI). (i) CRISPR/CAS9 guided cut at the predicted GOI/UTR boundary, (ii) a repair cassette containing the triple hemagglutinin epitope tag (3HA), the Chloramphenicol acetyltransferase (CAT) selection marker, and homology to the GOI/UTR boundary, is inserted between the GOI and UTR during cut repair guided by homology regions, (iii) GOI with the integrated repair cassette. The black arrows represent the four primers used to confirm integration via the PCRs shown in B. (B) Confirmation of generation of 3HA-tagging at the desired loci via PCR analysis using primers 1–4 (primers in S12 Table) shown in A. (TIF) [file ppat.1009301.s006.tif]

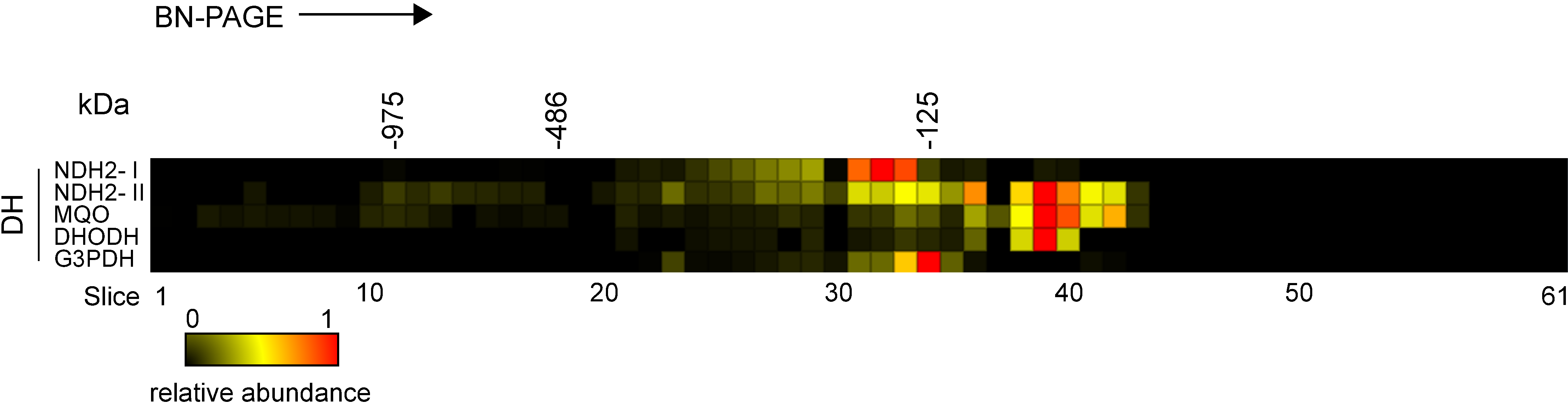

Supplement: S7 Fig — Heatmaps of the complexome profiles of additional Toxoplasma mitochondrial mETC dehydrogenases. Heatmaps represent the 61 gel slices the BN-PAGE gel was cut into, from the top (left) to the bottom (right) of the gel. Molecular weight markers shown on the top are based the migrations of mammalian mitochondrial complexes of known size. Protein names are shown on the left of their respective profiles. Red indicates the highest relative abundance (1) and black the lowest (0). (TIF) [file ppat.1009301.s007.tif]

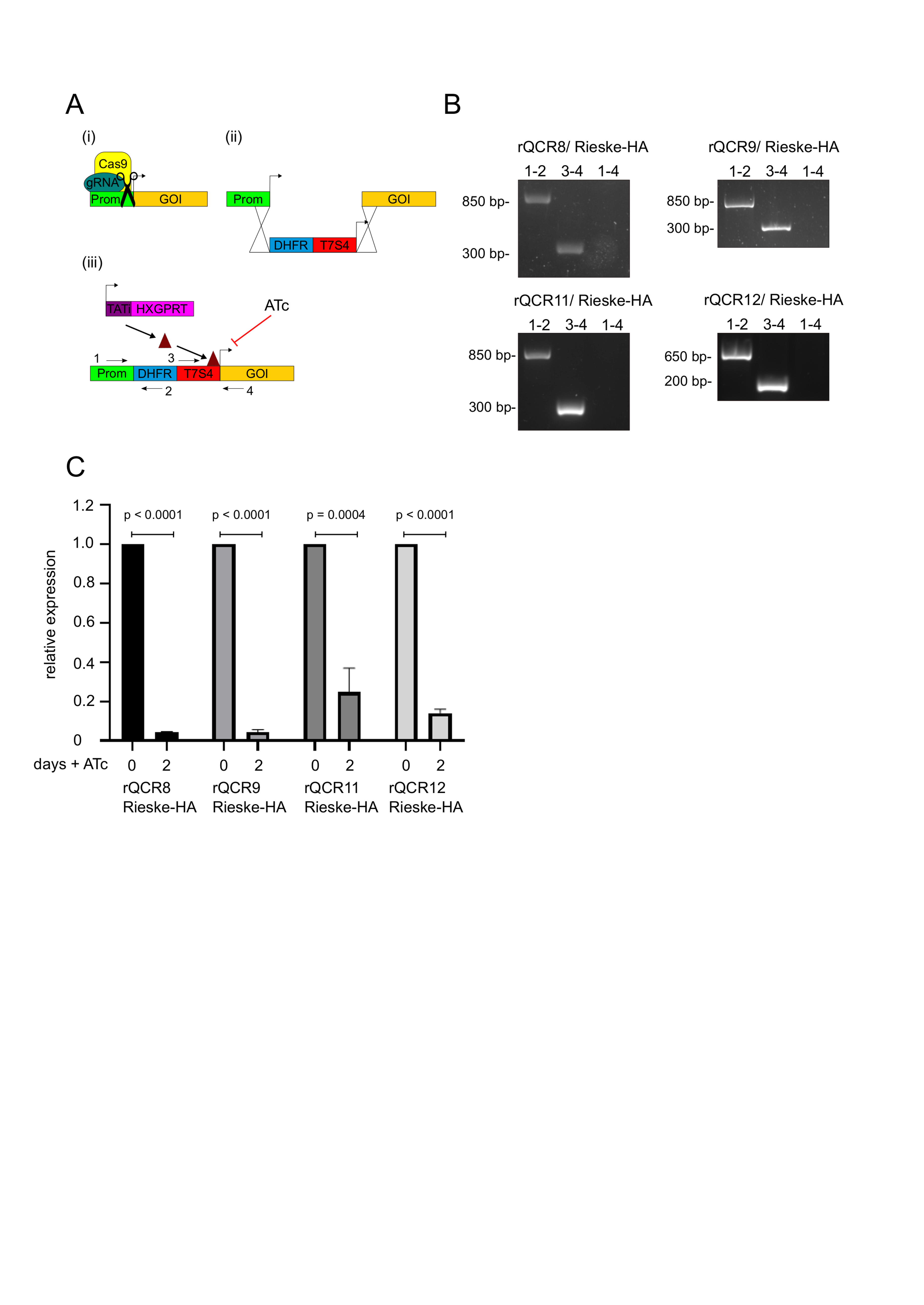

Supplement: S8 Fig — (A) Schematic depiction of the promoter replacement strategy allowing knock-down of a gene of interest (GOI) with the addition of anhydrotetracycline (ATc). (i) CRISPR/CAS9 guided cut at the predicted promoter/ATG boundary, (ii) a repair cassette containing the ATc repressible promoter, the dihydrofolate reductase (DHFR) selection marker, and homology to the promoter/ATG boundary, is inserted between the promoter and GOI during cut-repair guided by the homology sequences, (iii) GOI, under the control of ATc repressible promoter, is down regulated when ATc is added. The black arrows represent the four primers used to confirm integration via the PCRs shown in B (primers in S12 Table). (B) Confirmation of generation of promoter replacement at the desired loci via PCR analysis using primers 1–4 (primers in S9 Table) shown in A. (C) Transcript levels of each gene (QCR8,9,11,12) were analysed by qRT-PCR, in the absence (-) or presence (+) of ATc after 2 days. Bars represent the mean ± SEM (n = 3). (TIF) [file ppat.1009301.s008.tif]
