# Supplementary material for: Complexome profile of Toxoplasma gondii mitochondria identifies divergent subunits of respiratory chain complexes including new subunits of cytochrome bc1 complex
Source: PLoS Pathog. 2021 Mar 2;17(3):e1009301. doi: 10.1371/journal.ppat.1009301 (PMC7987180; doi:10.1371/journal.ppat.1009301)
Supplement: Attachment — Submitted filename: Maclean_respond2.pdf [file ppat.1009301.s022.pdf]

Dear Michael, Vern, Kasturi and Michael

We thank you and the reviewers for taking the time to make further suggestions to improve the manuscript, especially so close to the holiday season. We have made the suggested changes, as detailed below, which we hope addresses the remaining concerns and makes the proteomic data more readily accessible to the reader.

Part I - Summary

Reviewer #2: The authors have revised their prior submission to strongly address reviewer critiques and suggestions. I assume that the mass spectrometry data generated in this study will be made available via the ToxoDB interface, including individual peptides detected for each protein. Such information will be a valuable resource for the community.

The dataset will be available to the community of ToxoDB (we have discussed this with Omar Harb and are in the process of submitting the necessary information). Further, the complete proteomic dataset will also be deposited on the CEDAR (The ComplexomE profiling DAta Resource) database (https://www3.cmbi.umcn.nl/cedar/browse/).

Part II – Major Issues: Key Experiments Required for Acceptance

Reviewer #1: After carefully reviewing the mass spectrometry data, the reviewer realized one issue the authors might have missed to address. Among 842 proteins identified (Table S2), 381 had only one peptide. When the detection limit is low, how to differentiate a signal from a noise? Have the authors repeated this complexome profiling several times?

Peptide identification was performed using the Proteome Discoverer software with an FDR (False discovery rate) of 5% compared to a decoy database, allowing identification of peptides above background noise, with a degree of probabilistic confidence. The majority of *Toxoplasma* proteins (531 out of 913) were identified with more than one peptide. A confident identification can be based on a single peptide presenting with a high ions score in cases where the protein has few tryptic peptides. Notably, of the 76 proteins assigned to the mETC, ATP synthase and the dehydrogenase (i.e. those In Figures 2D and S7), 67 were identified from two or more peptides. Seven of the nine proteins identified by one peptide scoring below the threshold for 1% FDR rate allowing confident assignment. We expanded the explanation of this in the Materials and Methods section (lines 721 - 726). The only exceptions were TGGT1_257160 and Cytochrome b, which scored above the 5% threshold but not the stricter 1% FDR level. These exceptions have now been noted in the text (lines 212-213, 294-295).

The complexome profile was performed once to provide candidate proteins for validation by other techniques, such as protein tagging, localisation, native-PAGE, and co-immunoprecipitation. Complexome data should always be complemented by other technique to avoid drawing erroneous conclusions from artefactual association of proteins. Our complexome profile identifies many putative subunits of respiratory complexes, which are a starting point for further validation, as we did in the case of QCR8, QCR, QCR11 and QCR12.

Part III – Minor Issues: Editorial and Data Presentation Modifications

Reviewer #1: After carefully reviewing the revised paper, the reviewer still has several minor issues.
1, The complexome profiling data identified 842 Toxoplasma proteins in total, listed in table S2. The authors mentioned 264 of those are mitochondrial. Could the authors list these 264 proteins in a sperate excel table? Also, please list gene ID, protein annotation, peptide count, etc., in separate

excel columns. As it now, column A of S2 is hard to read with so much information in one cell (TGGT1:KE387283:30672:39285:-1 gene:TGGT1_243490:EPR56544 |putative BCS1 family isoform 9). Also, importantly, could the authors make the new table even more convenient to readers by designating some of the hypothetical proteins (in database) to known functions? At least, the authors have validated some unknown proteins in this study.

We have added a column to table S3 that lists mitochondrial proteins identified in our complexome profile. The list of mitochondrial proteins was made by comparing our complexome profile to the datasets of Seidi et al 2018 and Barylyuk et al 2020. Further, to increase the ease of readers looking at our proteomic data we have separated gene IDs and descriptions into different columns as the reviewer suggested, in table S2. We have also put in separate tables (Table S1A and S2A) the proteomic data used to create figures 2D and S7 - this makes it easier for the reader to find the data used to create complexome profiles of respiratory chain subunits. These tables also have the new annotations of hypothetical proteins that were discovered in the study, as suggested by the reviwer. The full proteomic datasets, of all discovered proteins, are still displayed below (Tables S1B and S2C).

2, Please list correct gene IDs of orthologs in other parasites in Table S7. The top hit IDs in table S7 are not ready for database searches, at least in a convenient manner.

The IDs in table S7 were the results from the HMMER search tool. We have added a column that displays these as gene IDs, to aide searches in other databases.

3, Legend of Table S1 lacks how the authors calculated abundance. How did the authors convert peptide counts to percentage of abundance? This math is critical to all analyses of this paper.

Details of the calculation of relative abundance are referred to in the materials and methods, where we also refer to a previous study from the lab (Bridges et al., 2017, “Subunit NDUFV3 is present in two distinct isoforms in mammalian complex I”). We now have added more text to this section to explain this process more clearly (lines 738 - 744).

Reviewer #3: - In respect to the SDHA homolog being not associated with CII in complexome: I thank the authors for sharing their IP data confirming association of SDHA with SDHB destined for another manuscript. I'm undecided if it is sufficient to state "IP experiments of complex II using either SDHB as bait consistently recover SDHA (Maclean et al in preparation).". The data availability of PLoS Pathogens (https://journals.plos.org/plospathogens/s/submission-guidelines) states that 'data not shown' is not an acceptable citation. If the editor agrees and if the other manuscript has been published or released as a preprint please replace with correct citation, otherwise remove. If removed I suggest the authors mention the caveat and suggest the IP as a method to confirm SDHA association with the complex in support of the other evidence cited in lines 526-548.

As suggested by the reviewer, we have removed this reference to unpublished data and have mentioned that future IP experiments should be performed to confirm SDHA’s association with complex II (lines 510 – 512).

- In respect to revised figure 2D I thank the authors for inclusion of the new names for CIII subunits. The revised discussed also renames a number of other subunits (e.g. TgCox4) so these should also be included in the figure for consistency.

Cox4, Cox6a and NDUFA4 have all been added to figure 2D.
