# Supplementary material for: Complexome profile of Toxoplasma gondii mitochondria identifies divergent subunits of respiratory chain complexes including new subunits of cytochrome bc1 complex
Source: PLoS Pathog. 2021 Mar 2;17(3):e1009301. doi: 10.1371/journal.ppat.1009301 (PMC7987180; doi:10.1371/journal.ppat.1009301)
Supplement: Attachment — Submitted filename: Maclean_PointByPoint.docx [file ppat.1009301.s021.docx]

Dear Michael, Vern, Kasturi and Michael

We would like to thank you and the reviewers for considering our manuscript for publication in PLOS pathogens and for providing insightful comments to improve it. In our revised manuscript, we had tried to address all the comments. Below is a point by point response, where text in black in the original text provided by the reviewers, text in *purple/italic* includes our explanations and responses, new additions to figures/tables in green and relevant lines in the manuscript revised in yellow.

**Reviewer #1: Major point:**

This study is carried out using the mitoribosomal tagged line (S35). Previously, this group has shown that mitoribosomal small subunit and large subunit were detected in BN-PAGE (Reference 34, Mol Micro 2019). Since proteomic approach of this study would detect every protein/complex from mitochondria, the authors need to show in separate tables or figures, the list of mitoribosomal proteins identified in this study.

*We now provide the list of mitoribosomal proteins identified in the gel slices analysed in details (Table S4). Only 3 proteins where identified in those slices, and their distribution does not indicate that they are a part of a complex that was resolved by our strategy (lines 153-155).*

*However, it is important to highlight that we chose to exclude the two top most slices of the gel from our in-depth proteomics analysis. This is due to previous experience indicating that these top slices contain high level of insoluble aggregates and complexes that could not fully enter the gel thus interfering with the per-slice-abundance analysis required for this method. We discuss this in the revised result section (lines 140-141).*

*We agree with the reviewer suggestion that this method should be explored to identify the mito-ribosome composition and we plan to develop the suitable conditions for this in the future.*

**Reviewer #2: Major points:**

1. The authors’ identification that N-terminal cyt c1 peptides co-migrate with complex V is interesting, but the analysis and interpretation here is unclear and somewhat confusing. Cyt c1 is expected to have an N-terminal mitochondrial-targeting sequence that is cleaved upon import, based on studies in yeast (Pubmed: 2822392). The authors hint at possible processing but do not discuss this literature and instead imply that the N-terminal leader sequence and cyt c1 domains may be separate proteins expressed from the same gene. A simple model is that the N-terminal targeting sequence is retained after cleavage from cyt c1 upon mitochondrial import. This model would be analogous to the N-terminus of mammalian Rieske that is retained as part of complex III after cleavage (Pubmed: 8386158).

*We thank the reviewer for pointing out this lack of clarity and for offering relevant literature. We revised this section to clarify the proposed model (which is indeed in line with the reviewer’s suggested model) and added the proposed reference (lines 541-544).*

Can the authors distinguish whether this N-terminal cyt c1 peptide is a stable subunit of complex V independent of interactions with complex III via the cyt c1 domain? For example, if the authors express an epitope-tagged copy of just the N-terminal ~150 amino acids of the cyt c1 protein, does it co-migrate with complex V by native PAGE and/or IP with complex V subunits? That experiment would substantially strengthen the very intriguing conclusion that this leader sequence associates with complex V independent of the cyt c1 domain.

*We agree with the reviewer that experimental evidence to reinforce the two independent proteins model is exciting. We now discuss two additional observations in support of this model (on top of the size of C-terminally tagged ATPTG1 that was described in the original manuscript):*

- *In all our Complex III immuno-precipitation (IP) experiments we only identify C-terminal peptides of TGGT1_246540 corresponding to the complex III subunit CytC1 portion of the protein, and not the ATP synthase subunit ATPTG1 portion.*
- *A separate work from our group, in collaboration with the Amunts lab, solved the structure of ATP synthase and showed that only the N-terminal amino-acids 32-153 encoded by TGGT1_246540 are included, corresponding to the ATPTG1 subunit. This work is in press and we hope to be able to include the citation in the current manuscript when it is accepted for publication.*

*We now discuss these observations in the discussion (lines 544-551) and include a Table S11 summarising our complex III IP attempts.*

2. The analysis and discussion of mitochondrial depolarization in figure 5B is confusing. (A) Is there prior published evidence that atovaquone treatment of *T. gondii* results in mitochondrial depolarization? The authors cite ref. 56 from *Plasmodium* as evidence that atovaquone depolarizes mitochondria, but follow-up studies in ref. 2 showed that ATV treatment alone does not depolarize the *P. falciparum* mitochondrion, due to the presence of a redundant, proguanil-sensitive proton-pumping pathway in blood-stage parasites thought to be the non-essential ATP synthase complex. *T. gondii* may differ in this regard from blood-stage *Plasmodium*, in which case inclusion of atovaquone in the figure 5B analysis would be helpful.

*We agree with the reviewer that the reference we used was not sufficient as the comparison between Plasmodium blood stages and Toxoplasma tachyzoites is not directly relevant. We now provide references for work performed directly in Toxoplasma tachyzoites where atovaquone treatment resulted in the ablation of membrane potential (references 58-59, line 452). We further include a control with atovaquone (new Figure 5B) and with the complex III inhibitor antimycin a. Both these inhibitors result in a clear decrease in red fluorescence, demonstrating that a defect in complex III function can be detected by this assay through decrease in red fluorescence.*

(B) Interpretation of figure 5B would be strengthened if the authors can also include a histogram plot of the fluorescence shifts upon each QCR knockdown, along with representative epifluorescence images of individual parasites.

*We now include data for all four QCR lines, and present as histogram plots (revised Figure 5B).*

*We find the fluorescence images un-informative: when the parasites are intracellular the host cell signal masks the parasites signal, and when extracellular and adhered to a cover slip the staining is not reproducible due to variability in parasite adherence and seeming unintended permeabilization of some adherent parasites.*

3. The authors present TOM40 detection in figure 6F as a negative control for specific loss of complex III signal upon knock-down of QCR proteins 8-12. The TOM40 signal is rather weak and the band position is not the same in all lanes, weakening the conclusion that QCR 8-12 knockdowns specifically destabilize complex III. Fig. 6 analysis would be strengthened by an additional negative control to rule out non-specific assembly defects due to general parasite malaise/death. For example, can the authors probe the blot in 6F for a different parasite protein (mitochondrial or cytosolic) that is not expected to be impacted by QCR knockdown, and/or treat parasites with a lethal inhibitor that targets something outside of the mitochondrion and show that ETC complex III stability is not impacted?

*We provide a new blot with TOM40 data where the signal and band position is improved (Revised Figure 6F). Likewise, we have added two additional controls to support the specific effect on complex III in the QCR mutants:*

1. *We include complex IV activity assay in native page, which shows that complex IV assembly is not affected either, thus providing an additional negative control to Tom40 antibody blot (Figure 6G).*
2. *We imaged and scored mitochondrial morphology. Defects in morphology are detected when mitochondrial biogenesis pathways are disrupted (e.g. as we showed in our DrpC mutant, PMID: 30947298), or when the parasites are dying (e.g. as we detected under non-specific effect of Cas9 over expression, PMID: 31339607, or after prolonged extracellular incubation, PMID: 28202940). In support of specificity, no significant change of mitochondrial morphology was observed upon QCRs depletion (Table S10).*

**Reviewer #3: No major issues**

**Reviewer #1: Minor points**

1, Introduction-depiction of the Q cycle is inaccurate. Ubiquinol (QH2) binds to the Qo site and donates electrons via two paths, heam groups on cyt b and the Rieske protein. At the Qi site, ubiquinone (Q) not ubiquinol (QH2) accepts the electron passed by cyt b. It becomes a semi-quinone at the first cycle of electron transport and ubiquinol at the second cycle of electron transport.

*This was corrected (line 94), we thank the reviewer for pointing out this mistake.*

2, Figure 2B, a typo of “totel”, a unnecessary “.” after the parenthesis. Supplementary Fig 8, “cat” repair should be cut repair. Page 13, Saccharomyces cerevisiae Cytochrome bc1, not b-c1. Page 32, Figure 5B legend, Valynomycin should be Valinomycin.

*These typos have been corrected (line 383 and Figure 5 legend), we thank the reviewer for pointing them out.*

3, Figure 2B, it looks like mitochondria are distributed in every fraction of the Percoll gradient, from top, 18/23 interphase to 23/40 interphase. With equal loading (5 ug), it seems that mitochondria are not enriched too much, although contaminants are largely reduced. Also, apicoplast co-exists with mitochondria throughout the gradient. It has been known that apicoplast might form contact sites with mitochondria. Although apicoplast contamination is expected, the authors should still mention that in the text (Results), in addition to Discussion.

*We now describe the outcome of our isolation of mitochondrial fraction in more detail in the result section including an added reference for apicoplast contamination (line 135-6).*

4, Figure S2A, it would be nice to add markers next to the gel to understand, roughly, the molecular weights of different complexes. S2B, this mass calibration shows complex V is smaller than 1000 kDa, isn’t it?

*We added a size marker to the revised FigureS2A now.*

*Note that the calibration in Former S2B (now Figure S2D) is for the mammalian complex, which is 538 kDa.*

5, Figure 2C legend, this doi link points to the mitochondrial genome paper, not the mitochondrial proteome paper.

*This was corrected, and we now use the peer reviewed reference, we thank the reviewer for pointing out this mistake.*

6, Supplementary data, which table is S2C, S2D?

*We mention each table that is linked to a figure in its corresponding figure legend. We also provided the supplementary tables data as excel file (rather than PDF) this time which we hope makes accessing the data easier.*

7, Figure S3, it would be nice to specify the sequences of truncated VS untruncated peptides underneath the profiles to help readers to understand how host peptides screw the detection of parasite peptides.

*We added alignments to the revised Figure S3 with the peptide sequence, where Common peptides between the two proteins are outlined in the alignment by a red box, and Toxoplasma specific peptides after truncation are marked by a blue box. Additionally, we have included the remaining Toxoplasma and Chlorocebus peptides after truncation in revised Table S6.*

8. Supplementary Fig. 5 legend, full names of Bb and Pf are missing.

*We thank the reviewer for pointing this out. These were added.*

9, Page 12. The doi link is for the mitochondrial genome paper, not the proteome paper. The same problem appeared again in Page 15.

*This was corrected, and we now use the peer reviewed reference, we thank the reviewer for pointing out this mistake*

10, Page 13. How did the author determine novel complex III subunits should be < 150 aa? It would be nice to show the sum of the molecular weights of 7 known subunits to indicate that estimation.

*This estimation was based on the size of the mammalian subunits for which homologs are missing in Toxoplasma, it is an hypothesis rather than a calculation. We thank the reviewer for pointing out that this wasn’t clear and we now clarify this point further (line 370-373).*

11, Page 32. Figure 5B, the numbers (% JC-1 positive cells) in the plots are invisible. Also, data of QCR11 should also be included in Figure 5B (albeit negative).

*We revised Figure 5B following one of the major comment above to show the shift in fluorescent populations as overlapping histograms, and we now also include all four lines.*

12, Page 33. Figure 6 legend, a “/” is missing between 9 and 11.

*This was corrected, we thank the reviewer for pointing out this mistake.*

13, Page 16. Discussion of complex II. It is interesting that the authors disputed two previous predicated membrane anchor proteins of complex II in Plasmodium (PfSDH3, Pf3D7_0611100; PfSDH4, Pf3D7_1010300). It still remains unknown which subunit proteins are membrane anchor proteins of complex II since neither the previous study (2009, reference 61) nor this study performed biochemical/biological analysis.

*We now discuss new observation from the literature that provides further evidence for the absence of these proteins from the Plasmodium complex and include reference to a new preprint describing a Plasmodium complexome (lines 628-632).*

Also, could be large molecular weight a result of super-complex formation with complex II and other complexes?

*In cases of super-complexes, other complex subunits would appear with high abundance together with complex II known and proposed units in the same gel slice. Our data indicates that the large size is formed with the identified subunits only.*

14, Page 19, is it a Percoll gradient or sucrose gradient? If it is Percoll, it should be 18/23/40, not 15/23/40.

*This was corrected, we thank the reviewer for pointing out this mistake.*

15, Page 20. It should be CytB, not COB.

*This was corrected, we thank the reviewer for pointing out this mistake.*

16, Readers should be able to download the individual excel sheets of Supplementary data when the paper is published.

*We agree with the reviewer. We had consulted PLOS pathogen’s editorial help about how to do upload the excel and it should now be provided with the current submission.*

**Reviewer #2: Minor points**

4. The manuscript title seems narrowly focused on complex III, even though important insights were obtained for other complexes. The authors may wish to re-word to more accurately reflect the breadth of insights provided by their study, for example “Complexome profile of Toxoplasma gondii mitochondria identifies divergent subunits of respiratory chain complexes.”

*We agree with the reviewer, but at the same time we do also wish to highlight the progress made with complex III – we thus propose the following title: “Complexome profile of Toxoplasma gondii mitochondria identifies divergent subunits of respiratory chain complexes including new subunits of cytochrome bc1 complex” (line 1-2).*

5. The extensive supplemental information is very helpful but lacks figure and table labels on each page and is thus difficult to navigate.

*We now added figure number in each figure and were relevant we mention the corresponding table in the figure legend.*

6. The authors name TGGT1_214250 and TGGT1_207170 as QCR11 and QCR12, respectively. These names differ from the names for the same genes assigned in the Hayward study. To avoid confusion, the authors of both papers should presumably agree on a common name for these subunits.

*We agree with this comment, we and have coordinated with Hayward and colleagues to unify the names which should appear in their upcoming publication in PLOS pathogens.*

7. The authors do not assess the degree of knockdown obtained +ATc for QCR proteins 8/9 and 11/12 in figures 5 and 6. Western blot analysis may not be possible if these proteins were not epitope-tagged, but the authors can at least assess mRNA reduction by RT-qPCR.

*qRT-PCRS showing and measuring the mRNA downregulation of each of the four QCRs are now included in Figure S8C.*

**Reviewer #3: Minor points**

 -Panel S2B should be changed to C, and C, D to B, C to reflect order discussed in text.

*The order of panels was aligned to appearance in the text in the revised Figure S2, we thank the reviewer for noting this.*

-Supplemental Tables (the large proteomics/informatics ones) were presented as excel files saved in the pdf format. I acknowledge that this could be as a result of the journal upload system and was not the intention of the authors. If this was intended please replace by actual excel (or csv) sheets as presentation in pdf format is not really useful to the reader since the formatting is lost (I found it very frustrating and just gave up on them, so this is a caveat in my review!).

*We fully agree with the reviewer (and indeed provided PDF due to our understanding of the instructions on the submission system). We had consulted PLOS pathogens editorial help about how to do upload the excel and it should now be provided with the current submission.*

-The authors often compare their complex mass estimates to other publications that used different detergents, most of which alter the migration of complexes on BN-PAGE either due to dissociation of subunits or possible assembly into supercomplexes (the latter is nicely illustrated in Fig 4F). While this is mentioned in the discussion, given the authors discovery of parasite supercomplexes that can be resolved using digitonin, and the fact that some of said other studies used digitonin or TX-100 in their size calculations (vs DDM for this study) I think it is fair to raise this caveat at the initial point of mention in the results.

*We now added a sentence highlighting this point upon the first mentioning of complex size difference from previous studies (ATP synthase) in the result section (lines 192-193).*

-A similar issue on mass estimates arises in the CII and III sections where the authors quote the digitonin resolved sizes of 130 kDa and 500 kDa (Schaegger 2000 EMBO J). Perhaps it is safer when referring to mammalian complexes to use the absolute calculations from Hirst, Kunji, Walker 2019 Science?

*We now added this reference (ref no 39, lines 165 and 335).*

-The CIII2+IV SC mass used in the authors calibration appears to be CIII2+IV2 (Table S4) though this isn’t reflected in the figure. Is this SC assembly stable in DDM? Please clarify and if no, revise calculations appropriately.

*In fact, here we do believe the mass is for CIII2 + IV, and we confirmed the correct values both in the figure and the table. The size, 693, corresponds to 2 x 243 (complex III) and 207 (complex IV). We detect subunits from mammalian complex III and IV in the same gel slice (slice 14), indicating that this particular supercomplex is at least partially stable in the experimental conditions used.*

-Are any of the new CIV (or III) subunits the homolog of NDUFA4, which is known to be present in P. falciparum (Balsa 2012 Cell Metab)?

*We thank the author for raising this important point. We now include a discussion about subunits with similarity to previously known proteins including NDUFA4 (lines 557-565) as well as updated the revised Table S9.*

-The text describes the SDHA subunit profile being split into two peaks due to some peptides being identical to the host SDHA peptides, similar as for ATPa/b subunits. In this case however, once considering only the parasite specific peptides the peak still co-migrated with the host complex (slice 35 vs slice 18 for the parasite). Does this mean the toxoplasma SDHA subunit isn’t associated with the other Complex II subunits (and possibly not a CII subunit at all)?

*We agree with the reviewer that this is a surprising finding, yet our data generated within a separate study of Complex II provide support for SDHA being a part of this complex. We performed 3 IP experiments using SDHB as bait and in all repeats we recover SDHA (while in contrast none of 3 IPs repeats with the complex III subunits CytC1 recovered SDHA, which provides a negative control). We provide a table summarising these data along with an image of the IP enrichment of the baits, for the reviewer’s discretion, and we mention this observation in the discussion (lines 578-581). Our Complex II study is performed in collaboration with another team, therefore we cannot share he data within the current publication.*

-There appears to be a typo “In addition to complex II, we observed evidence for the following quinone reductases (Figure 1B): two single-subunit type II NADH dehydrogenases (NDH2)…” on p12 since Figure 1B has nothing to do with this text.

*This was corrected, we thank the reviewer for pointing this out.*

-Putative CIII subunit (later ruled out) TGGT1_297160 is not listed in Table 2 though discussed extensively in text. Since the only other place to refer to it is the large Table S1 (which is rendered in an unreadable format) I think including it here plus indicating that it was excluded in the legend will be less confusing to readers.

*This gene is now mentioned in the revise Table 2 legend (in addition to all supplementary data now provided as Excel).*

-It would be helpful to include the new protein names (next to the original TGGT1_xxx) in parentheses on Fig 2B

*The new names are now included in the revised Figure 2D.*

-Regarding QCR7 and the conclusion that the low relative levels indicates that it may be expressed at a lower level. Can you exclude the HA tag impacting assembly into the complex and therefore leading to its turnover?

*We agree with the reviewer that the possibility of turnover induced through loss of complex III recruitment cannot be excluded. We mention this possibility too now (lines 421-422).*
